# A Fluorescence‐Based Assay for Screening β‐Lactams Targeting the *Mycobacterium tuberculosis* Transpeptidase Ldt_Mt2_

**DOI:** 10.1002/cbic.201900379

**Published:** 2019-11-08

**Authors:** Mariska de Munnik, Christopher T. Lohans, Gareth W. Langley, Corentin Bon, Jürgen Brem, Christopher J. Schofield

**Affiliations:** ^1^ Chemistry Research Laboratory University of Oxford 12 Mansfield Road Oxford OX1 3TA UK; ^2^ Department of Biomedical and Molecular Sciences Queen's University 18 Stuart Street Kingston ON K7L 3N6 Canada; ^3^ Present address: Charles River Laboratories Chesterford Research Park Saffron Walden Essex CB10 1XL UK; ^4^ Present address: Department of Structural Biology and Chemistry Institut Pasteur UMR 3523 CNRS Rue du Dr. Roux 75015 Paris France

**Keywords:** antibiotics, beta-lactams, fluorescent probes, inhibitors, tuberculosis

## Abstract

*Mycobacterium tuberculosis* l,d‐transpeptidases (Ldts), which are involved in cell‐wall biosynthesis, have emerged as promising targets for the treatment of tuberculosis. However, an efficient method for testing inhibition of these enzymes is not currently available. We present a fluorescence‐based assay for Ldt_Mt2_, which is suitable for high‐throughput screening. Two fluorogenic probes were identified that release a fluorophore upon reaction with Ldt_Mt2_, thus making it possible to assess the availability of the catalytic site in the presence of inhibitors. The assay was applied to a panel of β‐lactam antibiotics and related inhibitors; the results validate observations that the (carba)penem subclass of β‐lactams are more potent Ldt inhibitors than other β‐lactam classes, though unexpected variations in potency were observed. The method will enable systematic structure–activity relationship studies on Ldts, thereby facilitating the identification of new antibiotics active against *M. tuberculosis*.

## Graphical abstract

**Cysteine‐selective fluorogenic probes** enable the fluorescence‐based screening of inhibitors of Ldt_Mt2_, a transpeptidase antibiotic target in *M. tuberculosis*. The assay, which is amenable to high‐throughput screening, demonstrates the efficacy of the penem and carbapenem classes of β‐lactam antibiotics.

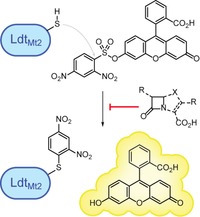

## Introduction

Inhibition of cell‐wall peptidoglycan biosynthesis has long been successfully exploited in the treatment of bacterial infections. β‐Lactams, the most widely used class of antibiotics, target peptidoglycan biosynthesis mainly through inhibition of d,d‐transpeptidases (or penicillin‐binding proteins; PBPs).^1^ In Gram‐negative bacteria, the transpeptidase domains of PBPs are responsible for the formation of 4→3 peptide crosslinks between *meso*‐diaminopimelate (*meso*‐Dap) and d‐alanine residues (i.e., *meso*‐Dap‐d‐Ala crosslinks) from two different peptide subunits in cell‐wall precursors.^2^ However, in mycobacteria such as *Mycobacterium tuberculosis*, the causative agent of tuberculosis (TB), the peptidoglycan layer contains high levels of 3→3 (*meso*‐Dap‐*meso*‐Dap) crosslinks, the formation of which is catalysed by l,d‐transpeptidases (Ldts).^2^

TB is the leading cause of death from a single infectious agent, and there is a pressing need to develop novel TB therapies.^3^ Ldt_Mt2_ from *Mycobacterium tuberculosis* appears to be of particular importance for virulence, as its loss leads to altered morphology and inhibition of colony growth.^4^ Certain β‐lactam antibiotics inhibit Ldt_Mt2_, in particular members of the (carba)penem subclass, and these represent potential leads for treatment of TB.^5^, ^6^, ^7^, ^8^, ^9^, ^10^ However, inhibitor discovery and development is severely limited by the current inhibition assays used for the Ldts.

Previously described low‐throughput assays for the Ldts have relied on methods such as mass spectrometry (MS), isothermal titration calorimetry (ITC), stopped‐flow fluorescence spectroscopy and hydrolysis of the chromophore‐containing β‐lactam nitrocefin.^4^, ^5^, ^6^, ^7^, ^8^, ^9^ In addition, as the Ldt_Mt2_ construct used for assays contains only one cysteine residue (i.e., Cys354, which is located in the active site, and is catalytically essential), the thiol‐reactive compound 5,5′‐dithiobis‐(2‐nitrobenzoic acid) (DTNB or Ellman's reagent) has been applied in colorimetric assays.^5^ Although potentially useful, these techniques are accompanied by limitations such as poor sensitivity and high protein requirements.^5^, ^6^ We were therefore interested in exploring the development of a high‐throughput fluorescence‐based assay for efficient screening of Ldt_Mt2_ inhibitors.

Inspired by the DTNB method,^5^ we considered the possibility of developing an assay based on the use of cysteine‐selective fluorogenic probes. With such an assay, the impact of inhibitors on the availability of the catalytic site could be tested through the (irreversible) reaction of the active‐site cysteine with a fluorogenic probe, providing a “nonclassical” inhibition assay. Cysteine labelling with fluorogenic compounds is a widely applied concept, but is often nonselective.^11^, ^12^ To our knowledge, no cysteine‐specific fluorogenic probes have been applied to the identification of competitive inhibitors for the Ldts. Herein, we report the development of an Ldt_Mt2_ assay based on the reaction of the active‐site cysteine with a fluorogenic reagent.

## Results and Discussion

### Selection of the fluorogenic reagent

**Scheme 1:**
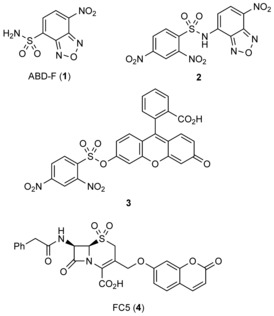
Structures of the fluorogenic probes investigated in this study.

A variety of thiol‐reactive fluorogenic compounds have been described that are either commercially available or that can be obtained through well‐defined synthetic steps.^13^ From these, ABD‐F (**1**), the benzoxadiazole probe **2** and the fluorescein probe **3** (Scheme 1) were selected and tested for reactivity with Ldt_Mt2_.^14^, ^15^, ^16^ As Ldt_Mt2_ covalently interacts with β‐lactam antibiotics, the fluorogenic β‐lactamase substrate FC5 (**4**; Scheme 1) was included in the screen.^17^

**Figure 1:**
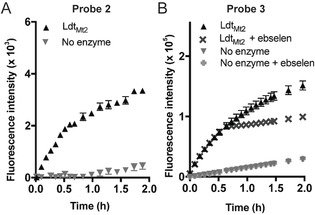
An increase in fluorescence intensity is observed on addition of fluorogenic probes **2** and **3** to Ldt_Mt2_. A) Probe **2** (*λ*_ex_=480 nm, *λ*_em_=555 nm) could be used at a concentration of 25 μm with 1 μm Ldt_Mt2_ to provide a sufficient level of fluorescent signal. B) Probe **3** (25 μm; *λ*_ex_=480 nm, *λ*_em_= 520 nm) showed better sensitivity than **2**, and provided a sufficient fluorescent signal with 100 nm Ldt_Mt2_. The reaction between Ldt_Mt2_ and **3** was quenched after 30 min by the addition of ebselen to allow for high‐throughput screening. Experiments with probe **2** were carried out in 50 mm sodium phosphate, pH 8.0, 0.01 % Triton X‐100, and those with probe **3** were in 50 mm HEPES, pH 7.2, 0.01 % Triton X‐100. Data points represent the mean, and error bars represent the standard deviation (*n*=96).

ABD‐F did not react in a sufficiently selective manner with Cys354 of Ldt_Mt2_ leading to a nonspecific increase in fluorescent signal that could not be related to the availability of the active‐site cysteine thiol (data not shown). FC5, which we have found to be a useful reporter for β‐lactamases,^17^ did not react efficiently with Ldt_Mt2_ (data not shown). Therefore, these potential probes were considered to be unsuitable for further assay development. However, an increase in the fluorescence signal was observed when Ldt_Mt2_ was treated with fluorogenic probes **2** and **3** (Figure 1).^15^, ^16^ Based on these promising results, subsequent experiments focused on optimising conditions for the use of **2** and **3**. The assay was more sensitive with **3** (*λ*_ex_=480 nm, *λ*_em_=520 nm), as the concentration of Ldt_Mt2_ could be lowered to 100 nm with a probe concentration of 25 μm (probably due to the high quantum yield associated with the fluorescein fluorophore). Probe **2** (*λ*_ex_=480 nm, *λ*_em_=555 nm) required an enzyme concentration of 1 μm with a probe concentration of 25 μm to obtain a sufficient signal window. Both **2** and **3** were observed to undergo nonenzymatic hydrolysis under the tested assay conditions that led to a linear increase in fluorescent signal independent of Ldt_Mt2_ (Figure 1).

### Optimisation of the assay with probes 2 and 3

We then investigated the optimal buffer conditions for **2** and **3**, with attention to minimising nonenzymatic hydrolysis, while maintaining the apparent reaction between the fluorogenic probes and the active‐site thiol of Ldt_Mt2_. Although the reaction between **3** and Ldt_Mt2_ proceeded rapidly in sodium phosphate‐based buffers, background hydrolysis of **3** in buffer alone was observed (Figures S1 and S2 in the Supporting Information). Alternatively, when the reaction was performed in 2‐[4‐(2‐hydroxyethyl)piperazin‐1‐yl]ethanesulfonic acid (HEPES) buffer, a satisfactory reaction rate was obtained for **2** and **3**, while keeping the nonenzymatic reaction of the probe at a low rate (Figures 1, S1 and S3). Sodium phosphate buffer best supported the reaction between **2** and Ldt_Mt2_, which showed a lower tendency to hydrolyse compared to **3** (Figure S3). Using elevated pH increased the reaction rate, while also increasing hydrolysis of the probe. Based on these experiments, the optimal balance between reaction rate and probe hydrolysis was found to occur at pH 8.0 for **2** (Figure S4) and pH 7.2 for **3** (Figure S5). Addition of NaCl to the buffer decreased probe degradation, but had a negative impact on reaction rate and was therefore omitted from subsequent assays (Figure S4). As metal ions (i.e., Cu^2+^) are thought to interact directly with Cys354 of Ldt_Mt2_,^18^ sodium ions in the buffer might similarly slow reaction with probes **2** and **3**.

The assay using **3** was considered suitable for endpoint assessment (Table 1). The optimal measurement time with this probe, identified based on *Z′* and signal to background (S/B) values, was found to be 30 minutes after reaction initiation. These conditions, which provided *Z′* and S/B values of 0.82 and 8.1, respectively, are likely to be suitable for high‐throughput screening (HTS). However, to permit HTS, methods for quenching the reaction were sought, and a panel of cysteine reactants was assessed for their ability to react with Ldt_Mt2_ (unpublished data).^19^ Ebselen, a known cysteine‐reactive reagent,^20^, ^21^, ^22^, ^23^ was found to rapidly quench the reaction between Ldt_Mt2_ and **3** (Figure 1 B). Due to continuous probe hydrolysis, the *Z′* and S/B values were decreased to 0.75 and 3.3, respectively, after 2 hours. An endpoint assay was not suitable for **2**, as the *Z′* and S/B values were inadequate when an enzyme concentration of 1 μm was used. By contrast, an assay based on kinetic analyses of the interaction of Ldt_Mt2_ with **2** yielded a *Z′* value of 0.77 and an S/B of 92.7 (Table 1).

**Table 1:** Signal to background ratio and *Z′* of **2** and **3** with Ldt_Mt2_.

Probe	Measurement	Time	*Z′* ^[a]^	S/B
3^[b]^	endpoint	30 min	0.82	8.1
3	endpoint	2 h	0.75	3.3
3	endpoint	4 h	0.62	2.1
3	kinetic	30 min	0.84	9.0
2	endpoint	30 min	0.44	14.3
2	kinetic	30 min	0.77	92.7

[a] Z′ was calculated by using the formula *Z′*=1−(3(*σ*_p_+*σ*_n_))/|*μ*_p_−*μ*_n_|(*σ*=standard deviation, *μ*=mean, p=positive control, n=negative control; *n*=96). The positive controls consisted of Ldt_Mt2_ (1 μm or 100 nm, for probes **2** and **3**, respectively) with probe **2** or **3** (25 μm or 100 nm, respectively); the negative control was the probe alone. [b] For endpoint measurements with probe **3**, ebselen was added at *t*=30 min, and measurements were made at the indicated time.

### Mass spectrometric analysis of the reaction between Ldt_Mt2_ and probes 2 and 3

**Scheme 2:**
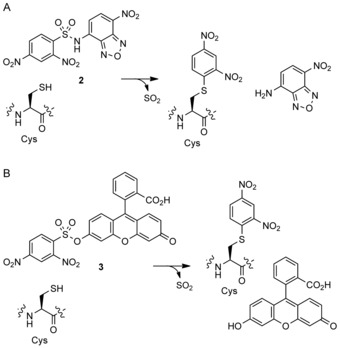
Schematic representation of the reactions between Ldt_Mt2_ and A) **2** or B) **3** releasing benzoxadiazole and fluorescein fluorophores, respectively.

To gain insight into the mechanism of the reaction between the fluorogenic probes and Ldt_Mt2_, we performed MS analyses. For both **2** and **3**, a single adduct was observed upon incubation with Ldt_Mt2_ under standard conditions, corresponding to a mass increase of 169 Da (Figure S6). These results suggest that a single molecule of **2** or **3** reacts with Ldt_Mt2_ leading to the arylation of the cysteine residue with the dinitrophenyl group, and the release of SO_2_ and the fluorophore (Scheme 2). MS analyses showed the obtained adduct to be stable for at least 24 hours (data not shown).

### Inhibition of Ldt_Mt2_ by β‐lactam antibiotics and related inhibitors

**Figure 2:**
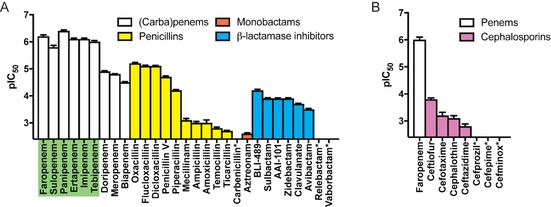
pIC_50_ values for Ldt_Mt2_ inhibition by a selection of (carba)penems, penicillins, cephalosporins, monobactams, and β‐lactamase inhibitors were obtained by using the fluorogenic assay with A) probe **3** and B) probe **2**. Data points represent the mean; error bars represent the standard deviation (*n*=4). For inhibitors indicated with asterisks, the pIC_50_ values were too low to be measured with the assay (pIC_50_<2.4). The most potent inhibitors (pIC_50_>5.5) are highlighted in green. Note that ebselen was not used to quench the interaction between Ldt_Mt2_ and probe **3** in these experiments.

With the conditions optimised, the assay was then applied to analyse the inhibition of Ldt_Mt2_ by 28 β‐lactam antibiotics, including penems, carbapenems, penicillins, cephalosporins and monobactams, in addition to eight known β‐lactamase inhibitors (Figures 2 and S7). As probe **3** could be used in endpoint assays with lower enzyme concentrations, increasing the throughput of the assay, we focused on probe **3** for screening. Although satisfactory data were acquired for most classes of β‐lactams and β‐lactamase inhibitors when using probe **3**, apparent background reactions of cephalosporins with **3** were observed. At high cephalosporin concentrations, the measured fluorescent intensities were greater than would be expected for the complete reaction with Ldt_Mt2_ with **3**, thus precluding the determination of inhibitor potency with this probe (data not shown). It is unclear whether this background reaction is due to the direct reaction of cephalosporins with probe **3**, or if cephalosporin degradation products might instead be responsible. Nevertheless, this background reaction was not observed between cephalosporins and probe **2**, and thus probe **2** was used to assay the inhibition of Ldt_Mt2_ by β‐lactams belonging to this class.

In agreement with previous reports, the penems and carbapenems were found to be the most active class of β‐lactams inhibiting Ldt_Mt2_, followed by the penicillins (Figure 2 A).^5^, ^6^ In particular, the penems faropenem and sulopenem were among the most potent inhibitors of Ldt_Mt2_. Interestingly, the tested members of both the carbapenem and penicillin classes show considerable variations in potency; this probably relates to the substantial differences in their side chains (Table S1). Whereas the carbapenems panipenem, tebipenem, imipenem and ertapenem showed similar activities to the penems, doripenem, meropenem and biapenem were less potent. The penicillins oxacillin, flucloxacillin, dicloxacillin, penicillin V and piperacillin all had a significant inhibiting effect on the enzyme, whereas mecillinam, amoxicillin, ampicillin, temocillin, ticarcillin and carbenicillin showed little to no inhibition (Figure 2 A). The seven cephalosporins tested (as well as the lone monobactam, aztreonam) showed relatively poor inhibition of Ldt_Mt2_ (Figure 2), although a similar side‐chain dependence was observed, as occurred with the penicillins. The overall trends observed here are in general agreement with those reported previously for some of these β‐lactams with Ldt_Mt2_ using nitrocefin hydrolysis and DTNB assays.^5^, ^6^

The outcomes of the interactions between some β‐lactams and Ldts can be complex, thus the preliminary inhibition data reported here merit further investigation. Rapid fragmentation has been observed for the covalent complexes derived from Ldt_Mt2_ with the penems faropenem and sulopenem; this might contribute to their potency as Ldt_Mt2_ inhibitors.^5^, ^6^, ^24^ Although an analogous reaction also occurs with some penicillins, the rate of fragmentation appears to depend on the penicillin side chain.^24^ However, it is currently unclear how this fragmentation relates to the inhibitory potency of the penicillins with Ldt_Mt2_.

Several serine β‐lactamase inhibitors, including those used clinically, showed moderate levels of Ldt_Mt2_ inhibition (Figure 2 A). The 6‐alkylidenepenem sulfone BLI‐489 showed the greatest inhibitory activity, whereas penicillin sulfones (sulbactam, AAI‐101) and clavulanic acid demonstrated a small decrease in potency. Variations were observed between the different diazabicyclooctane (DBO)‐type inhibitors tested; moderate inhibition was observed for zidebactam and avibactam, whereas relebactam did not inhibit Ldt_Mt2_ at the levels tested. The targeting of Ldts by DBO inhibitors has been observed in previous work showing that avibactam potentiates the activity of the penicillin amoxicillin against *M. tuberculosis*.^25^ Interestingly, the cyclic boronate β‐lactamase inhibitor vaborbactam^26^ did not inhibit Ldt_Mt2_ under these conditions, potentially due to the different interactions of active‐site serine and cysteine residues with boron.

## Conclusion

In conclusion, a fluorescence‐based assay for Ldt_Mt2_ with cysteine‐selective fluorogenic probes **2** and **3** has been developed. The optimised endpoint assay with probe **3** is amenable to high‐throughput screening, with S/B>8 and *Z′*>0.8. Although the assay with probe **2** is less sensitive than that for **3**, probe **2** was observed to be more hydrolytically stable, and can be used in situations in which **3** reacts with inhibitors. Assays with **2** are therefore a useful tool to identify high‐quality hits during high‐throughput screening. The assays reported here for Ldt_Mt2_ may be applied to screening inhibitors of other cysteine‐containing enzymes, such as other Ldt subtypes and cysteine proteases, for example, cathepsins.

The assays were used to screen a library of β‐lactam and related antibiotics as well as several β‐lactamase inhibitors. The results reveal that, of the β‐lactam antibiotics tested, penems and carbapenems are the most potent classes of inhibitors of Ldt_Mt2_. Together with high‐throughput screening, this fluorogenic assay should help to identify new chemical templates for covalently reacting, mechanism‐based Ldt inhibitors, including β‐lactams optimised for the treatment of TB. The methods developed will also facilitate ongoing investigations on the different reactivities of transpeptidases employing a nucleophilic serine (i.e., PBPs) or a cysteine (i.e., Ldts).^24^ This work could ultimately lead to the identification of new antibiotics with increased potency against *M. tuberculosis* that target both Ldts and PBPs.

## Experimental Section

**Fluorogenic assay optimisation**: Reaction of Ldt_Mt2_ with **2** or **3** (at the indicated concentrations) was conducted in the indicated buffers on a 25 μL scale in 384‐well μ‐clear plates (clear bottomed, Greiner Bio‐One, part number 781096). Measurements involving **2** were made by using a BMG Labtech CLARIOstar microplate reader, with *λ*_ex_=480 nm and *λ*_em_=555 nm, with bottom optic reading, a focus of 3.5 mm and a gain of 1000. Measurements involving **3** were made on a BMG Labtech PHERAstar FS instrument, with *λ*_ex_=480 nm and *λ*_em_=520 nm, with bottom optic reading, a focus of 3.6 mm and a gain of 812.

**Fluorogenic assays with probe 2**: Assay buffer (14 μL, 50 mm sodium phosphate, pH 8.0, 0.01 % (*v*/*v*) Triton X‐100) was added to a black polystyrene, flat‐bottomed 384‐well μ‐clear plate (clear bottomed, Greiner Bio‐One, part number 781 096) by using a MultiDrop Combi reagent dispenser (Thermo Fisher). Inhibitor (1 μL) was added by using a CyBio liquid handling system (Analytik Jena AG, Germany). Ldt_Mt2_ (5 μL, final concentration 1 μm) was added, and the mixture was incubated for 10 min. Fluorogenic probe **2** (5 μL, 25 μm) was added, and the fluorescence signal was measured by using a BMG Labtech CLARIOstar instrument with *λ*_ex_=480 nm and *λ*_em_=555 nm. Readings were taken over a period of 30 min, at intervals of 60 s by using the bottom optic, with a focus of 3.5 mm and a gain of 1000. All reactions were carried out in quadruplicate, and controls without inhibitor and without Ldt_Mt2_ were included. The increase in fluorescence intensity within the initial linear range was calculated for each condition by using the SLOPE function of Microsoft Excel. The values were normalised against the mean average of no‐inhibitor controls and the mean average of no‐enzyme controls. The dose–response analysis was performed by using the log(inhibitor) versus normalized response− variable slope model in Prism (GraphPad). Data points were plotted as mean average with standard deviation as the error bars.

**Fluorogenic assays with probe 3**: Assay buffer (14 μL 50 mm HEPES, pH 7.2, 0.01 % (*v*/*v*) Triton X‐100) was added to a black polystyrene, flat‐bottomed 384‐well μ‐clear plate (clear bottomed, Greiner Bio‐One, part number 781096) by using a MultiDrop Combi (ThermoFisher). Inhibitor (1 μL) was added by using a CyBio liquid handling system (Analytik Jena AG, Germany). Ldt_Mt2_ (5 μL, 100 nm) was added, and the mixture was incubated for 10 min. Fluorogenic probe **3** (5 μL, 25 μm) was then added, and the plate was incubated for an additional 30 min. For high‐throughput applications, ebselen (100 μm) was added to quench the interaction between Ldt_Mt2_ and probe. Note that ebselen was not used for the experiments shown in Figure 2. The fluorescence signal was measured by using a BMG Labtech PHERAstar FS microplate reader with *λ*_ex_=480 nm and *λ*_em_=520 nm. The reading was taken by using the bottom optic, with a focus of 4.2 mm and a gain of 870. All reactions were carried out in quadruplicate, and controls without inhibitor and without Ldt_Mt2_ were included. The data were analysed by using Prism (GraphPad). Data points were plotted as mean average with standard deviation as the error bars. The values were normalised against the mean average of no‐inhibitor controls and the mean average of no‐enzyme controls. The dose–response analysis was performed by using the log(inhibitor) vs. normalized response−variable slope model in Prism (GraphPad).

**Mass spectrometry**: Protein mass spectra of the Ldt_Mt2_ adducts formed with fluorogenic probes **2** and **3** were obtained on a Waters LCT (TOF) system. Samples consisted of Ldt_Mt2_ (1 μm) and the fluorogenic probe **2** or **3** (100 μm) in sodium phosphate (50 mm, pH 7.5) and were measured after incubation periods of 5 min and 24 h.

## Conflict of interest

*The authors declare no conflict of interest*.

## Supporting information

As a service to our authors and readers, this journal provides supporting information supplied by the authors. Such materials are peer reviewed and may be re‐organized for online delivery, but are not copy‐edited or typeset. Technical support issues arising from supporting information (other than missing files) should be addressed to the authors.

Supplementary
