# Supplementary material for: A Fluorescence‐Based Assay for Screening β‐Lactams Targeting the Mycobacterium tuberculosis Transpeptidase LdtMt2
Source: Chembiochem. 2019 Nov 8;21(3):368–72. doi: 10.1002/cbic.201900379 (PMC7028133; doi:10.1002/cbic.201900379)
Supplement: Supplementary file 1 — Supplementary [file CBIC-21-368-s001.pdf]

CHEMBIOCHEM

Supporting Information

A Fluorescence-Based Assay for Screening β-Lactams Targeting the *Mycobacterium tuberculosis* Transpeptidase Ldt_{Mt2}

Mariska de Munnik,[a] Christopher T. Lohans,*[a, b] Gareth W. Langley,[a, c] Corentin Bon,[a, d] Jürgen Brem,[a] and Christopher J. Schofield*[a]

cbic_201900379_sm_miscellaneous_information.pdf

Author Contributions

M.d.M. Investigation: Lead; Writing - Original Draft: Equal
C.L. Investigation: Supporting; Supervision: Supporting; Writing - Original Draft: Supporting; Writing - Review & Editing: Lead
G.L. Investigation: Supporting; Supervision: Supporting; Writing - Review & Editing: Supporting
C.B. Investigation: Supporting
J.B. Funding acquisition: Equal; Supervision: Supporting; Writing - Review & Editing: Supporting
C.S. Funding acquisition: Equal; Supervision: Lead; Writing - Review & Editing: Equal.

Contents

Experimental Details S3

Figure S1. Effect of different buffers on the non-enzymatic breakdown of **3**. S5

Figure S2. Effect of different buffers on the reaction of Ldt_{Mt2} with probe **3** S6

Figure S3. Effect of different buffers on the reaction of Ldt_{Mt2} with probe **2**. S7

Figure S4. Effect of pH and NaCl on the reaction between probe **2** and Ldt_{Mt2}. S8

Figure S5. Effect of pH and NaCl on the reaction between probe **3** and Ldt_{Mt2}. S9

Figure S6. Mass spectra showing the reaction of Ldt_{Mt2} with **2** and **3**. S10

Figure S7. Dose-response curves for inhibitors with Ldt_{Mt2}. S11

Table S1. IC_{50} values for inhibitors with Ldt_{Mt2}. S13

References S17

Experimental Details

General

Chemicals were from Sigma Aldrich unless otherwise specified. Faropenem was from Selleckchem, doripenem and ertapenem were from Glentham Life Science, and meropenem, imipenem and aztreonam were from Molekula. Chemicals were used without further purification, unless otherwise stated. Column purifications were carried out using a Biotage Isolera One system unless otherwise specified. Thin-layer chromatography (TLC) was used to monitor reaction progress, and was performed using TLC silica gel 60 F254 sheets from Merck. NMR characterisation of synthesised compounds was performed using a 2-channel Bruker AVIIIHD 400 MHz nanobay instrument. High-resolution mass spectrometry (HRMS) characterisation was performed using a Thermo Exactive machine coupled with a UPLC system.

Protein production and purification

Ldt_{Mt2} Δ1-55 was produced and purified as described.[1] Briefly, a culture of *Escherichia coli* BL21(DE3) transformed with pNIC28-Bsa4-Ldt_{Mt2} Δ1-55 was grown in 2TY media (with 50 µg/mL kanamycin). Protein production was induced when the culture OD_{600} reached 0.6 through the addition of 0.5 mM isopropyl β-D-thiogalactopyranoside (IPTG). The culture was incubated at 18 °C, 180 rpm overnight. Ldt_{Mt2} Δ1-55 was purified using a 5 mL HisTrap column (GE Life Sciences), followed with a 300 mL Superdex 200 column (GE Life Sciences). Purified Ldt_{Mt2} Δ1-55 (in 50 mM Tris, pH 8.0, 100 mM NaCl) was frozen on liquid nitrogen and stored at -80 °C. The purity and identity of Ldt_{Mt2} Δ1-55 was confirmed by SDS-PAGE (>95 % purity) and mass spectrometry (calculated 40,444 Da, observed deconvoluted mass 40,441 Da).

(I) 7-Nitrobenzo[c][1,2,5]oxadiazol-4-amine (5)[2]

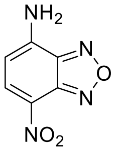

The reaction compounds were shielded from light during all stages of the synthesis. To a solution of 4-chloro-7-nitrobenzofurazan (500 mg, 2.50 mmol, 1.0 eq) in methanol (40 mL), NH_4OH (5.0 M in H_2O, 15.4 mL, 77.0 mmol, 30.8 eq) was added at room temperature. The resulting mixture was stirred for 6 hours. Solvents were evaporated under reduced pressure. The crude product was purified using column chromatography (cHex/EtOAc 100:0 to 0:100, 2x). **5** was obtained as a brown solid (0.320 g, 72%). ^{1}H NMR (400 MHz, DMSO-d6) δ 8.87 (br s, 2H), 8.50 (d, J = 8.8 Hz, 1H), 6.40 (d, J = 8.8 Hz, 1H). HRMS (ESI$^-$); $C_6H_3O_3N_4$ [M-H]$^-$; *m/z* observed 179.02092, calculated 179.02106.

(II) 2,4-Dinitro-N-(7-nitrobenzo[c][1,2,5]oxadiazol-4-yl)benzenesulfonamide (2)[2]

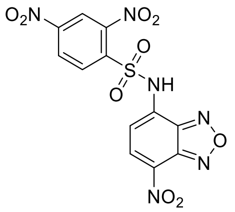

The reaction components were shielded from light during all stages of the synthesis. A solution of **5** (179 mg, 1.00 mmol, 1.0 eq) in anhydrous THF (25 mL) was cooled to 0 °C. NaH (60% in mineral oil, 57.4 mg, 1.56 mmol, 1.5 eq) was added. The resulting mixture was stirred for 15 minutes at 0 °C. 2,4-Dinitrobenzensulfonyl chloride (693 mg, 1.56 mmol, 1.5 eq) dissolved in anhydrous THF (5 mL) was added dropwise. The reaction mixture was allowed to warm to room temperature, then stirred for 2.5 h. The reaction mixture was cooled to 0 °C, and saturated $NaHCO_3$ (40 mL) was slowly added. The resulting mixture was extracted with EtOAc (30 mL, 3x). The combined organic layers were washed with brine (40 mL), dried with magnesium sulphate, then filtered. Solvents were evaporated under reduced pressure. The crude product was purified using column chromatography (cHex/EtOAc 100:0 to 30:70). **2** was obtained as a dark red solid (0.323 g, 76%). ^{1}H NMR (400 MHz, Methanol-d_4) δ 8.60 – 8.54 (m, 1H), 8.49 (dd, J = 8.5, 2.0 Hz, 1H), 8.38 (t, J = 9.0 Hz, 2H), 6.82 (d, J = 9.0 Hz, 1H).^{13}C NMR (400 MHz, Methanol-d_4) δ 151.12, 150.70, 150.39, 149.75, 145.64, 142.33, 137.15, 132.69, 127.10, 124.98, 120.58, 109.85. HRMS (ESI$^+$); $C_{12}H_5O_9N_6{}^{32}S$ [M+H]$^+$; *m/z* observed 408.98426, calculated 408.98442.

2-(6-(((2,4-Dinitrophenyl)sulfonyl)oxy)-3-oxo-3H-xanthen-9-yl)benzoic acid (3)[3]

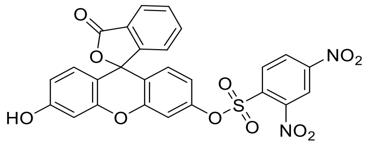

The reaction compounds were shielded from light during all stages of the synthesis. Fluorescein (665 mg, 2.00 mmol, 1.0 eq) was suspended in DCM (20 mL), then the temperature was reduced to 0 °C. Triethylamine (0.340 mL, 2.40 mmol, 1.2 eq) was added. The reaction mixture was stirred for 5 minutes, then 2,4-dinitrobenzenesulfonyl chloride (533 mg, 2.40 mmol, 1.2 eq) was added. The reaction mixture was allowed to warm to room temperature then stirred for 5.5 h. DCM (60 mL) was added and the mixture was washed with HCl (1.0 M, 60 mL, 3x) and brine (60 mL). The organic layer was dried over magnesium sulphate, filtered and solvents were evaporated under reduced pressure. The crude product was purified using column chromatography (cHex/EtOAc 100:0 to 60:40, 2x). **3** was obtained as a yellow solid (0.558 g, 50%). ^{1}H NMR (400 MHz, DMSO-d_6) δ 10.35 – 10.16 (m, 1H), 9.18 – 8.88 (m, 1H), 8.65 – 8.48 (m, 1H), 8.37 (d, J = 8.6 Hz, 1H), 8.12 – 7.97 (m, 1H), 7.89 – 7.71 (m, 2H), 7.45 – 7.31 (m, 2H), 7.07 – 6.86 (m, 3H), 6.76 – 6.56 (m, 2H). ^{13}C NMR (400 MHz, DMSO) δ 168.61, 168.36, 160.00, 155.84, 152.35, 151.79, 151.56, 151.43, 150.88, 149.59, 149.42, 148.28, 136.10, 133.79, 130.90, 130.65, 130.56, 130.45, 129.93, 129.31, 127.89, 125.77, 125.40, 125.10, 124.21, 122.14, 121.38, 121.20, 119.39, 118.80, 118.65, 117.90, 116.72, 113.59, 111.08, 110.92, 109.04, 102.47, 81.59, 80.07. HRMS (ESI$^+$); $C_{26}H_{15}O_{11}N_2{}^{32}S$ [M+H]$^+$; *m/z* observed 563.03920, calculated 563.3911.

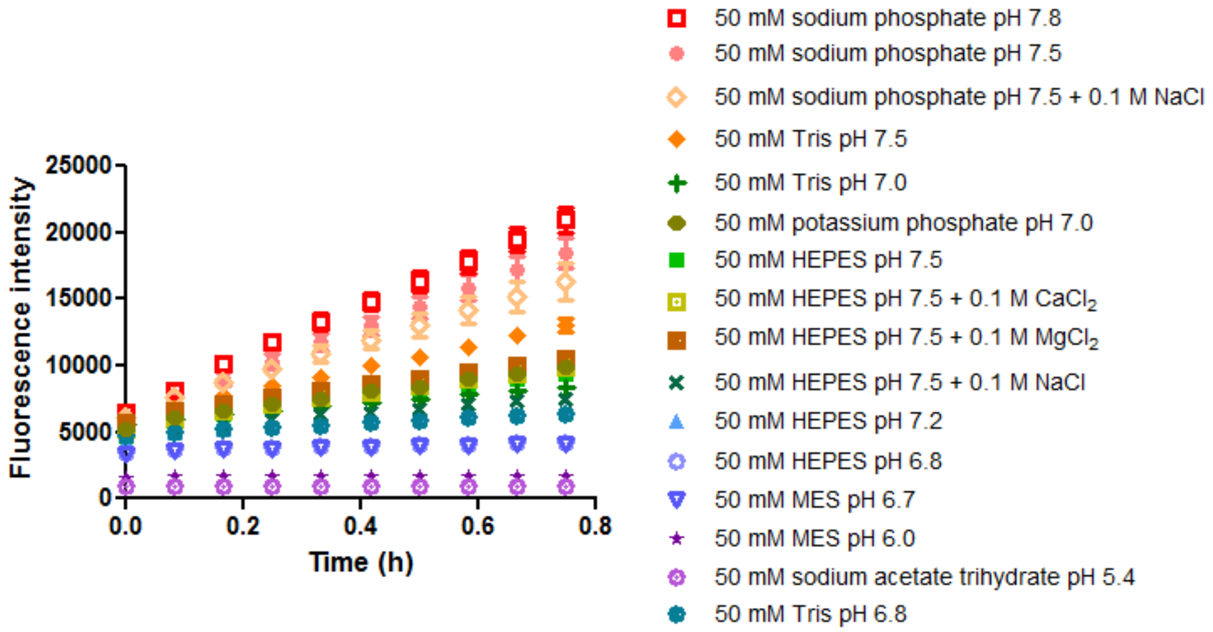

Figure S1. Impact of different buffers on the non-enzymatic breakdown of **3** (25 µM). Use of HEPES buffer led to a lower degree of degradation of **3**, compared to sodium phosphate or Tris at the same pH (7.5). Lowering the pH also led to reduced degradation of probe **3**. The data points shown represent the mean, with error bars representing the standard deviation (n = 24). See Experimental Details for the conditions used.

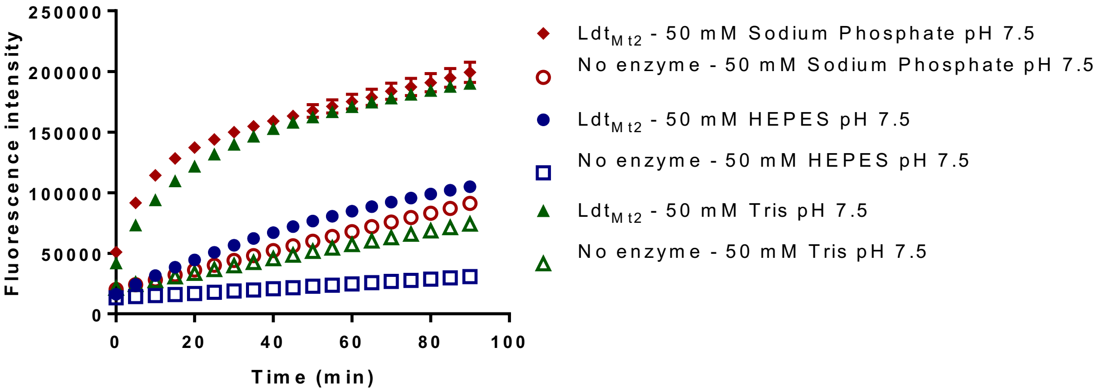

Figure S2. Impact of altering buffer conditions on the reaction of Ldt_{Mt2} (100 nM) with probe **3** (25 µM). Phosphate buffer and Tris buffer are both accompanied with significant non-enzymatic breakdown. In HEPES buffer, Ldt_{Mt2} and probe **3** react at a lower velocity, which is accompanied with a lower degree of degradation of **3**. All buffers contained 0.01% (v/v) Triton X-100. Data represents average and error bars represent standard deviation (n = 48).

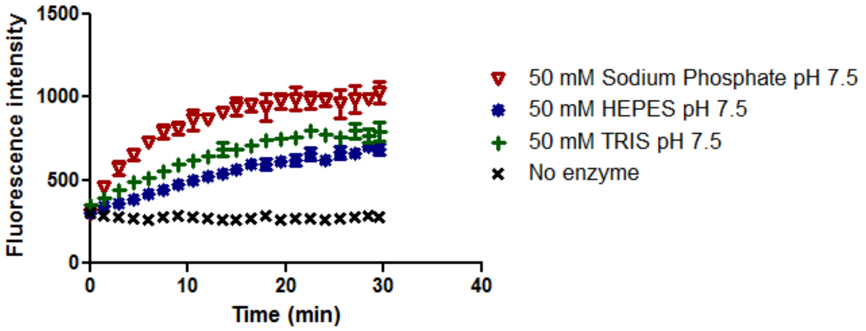

Figure S3. Impact of buffer conditions on the reaction of Ldt_{Mt2} (5 µM) with probe **2** (125 µM). Replacing phosphate buffer with HEPES or Tris significantly decreased the reaction of **2** with Ldt_{Mt2}. All buffers contained 0.01% (v/v) Triton X-100. The data points shown represent the mean, with error bars representing the standard deviation (n = 24). See Experimental Details for the conditions used.

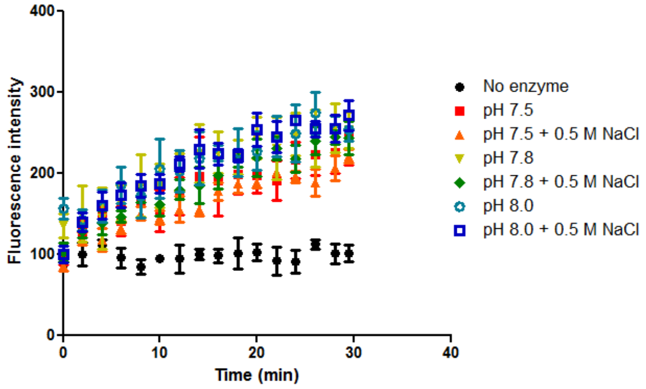

Figure S4. Impact of pH and NaCl on the reaction of Ldt_{Mt2} (1 µM) with probe **2** (25 µM) in 50 mM sodium phosphate buffer. The data points shown represent the mean, while the error bars represent standard deviation (n = 24). See Experimental Details for the conditions used.

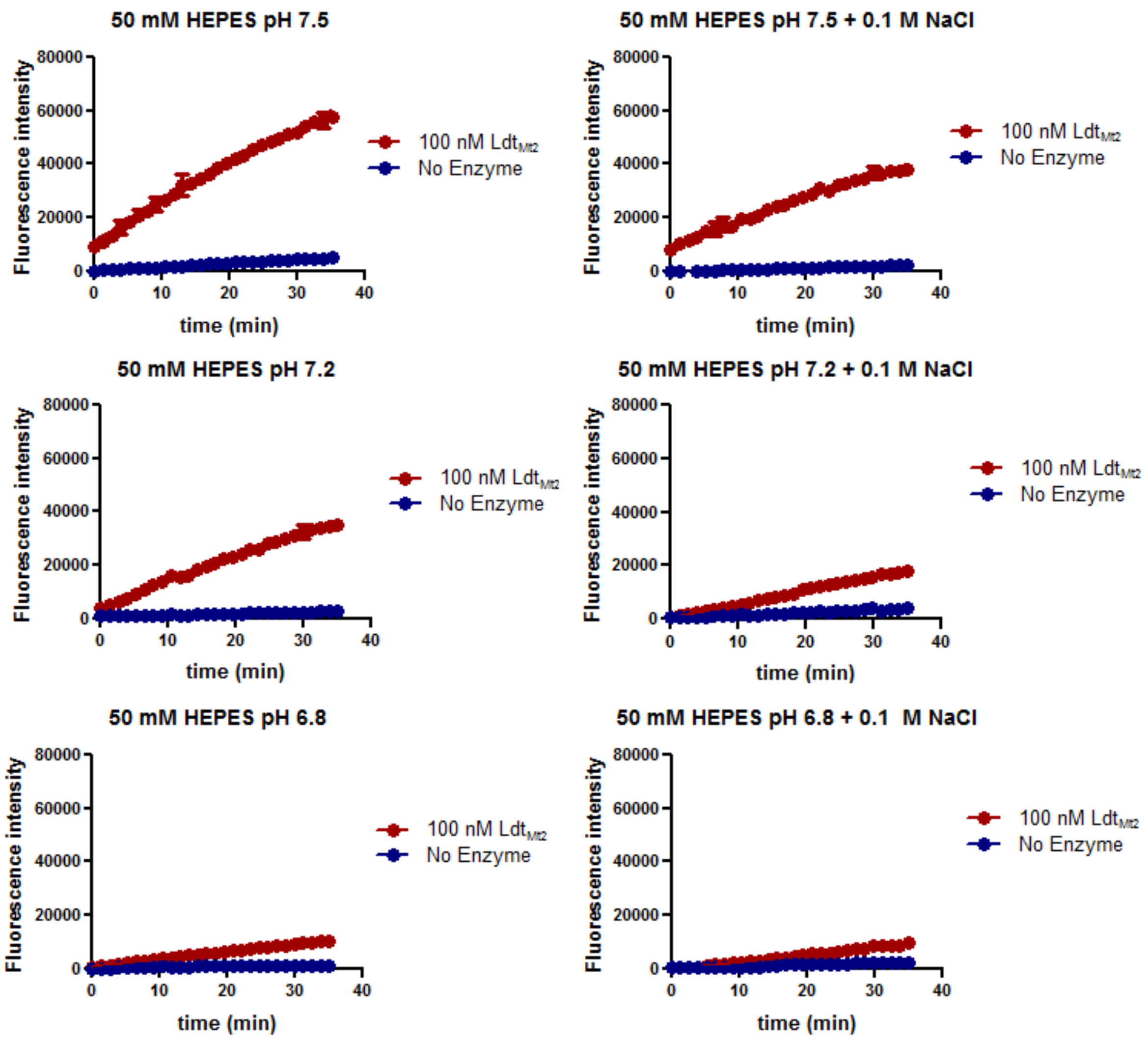

Figure S5. Impact of pH and NaCl on the reaction between probe **3** and Ldt_{Mt2} in 50 mM HEPES buffer. Lowering the pH and addition of NaCl both decreased the reaction rate. The data points shown represents the mean, while the error bars represent standard deviation (n = 24). See Experimental Details for the conditions used.

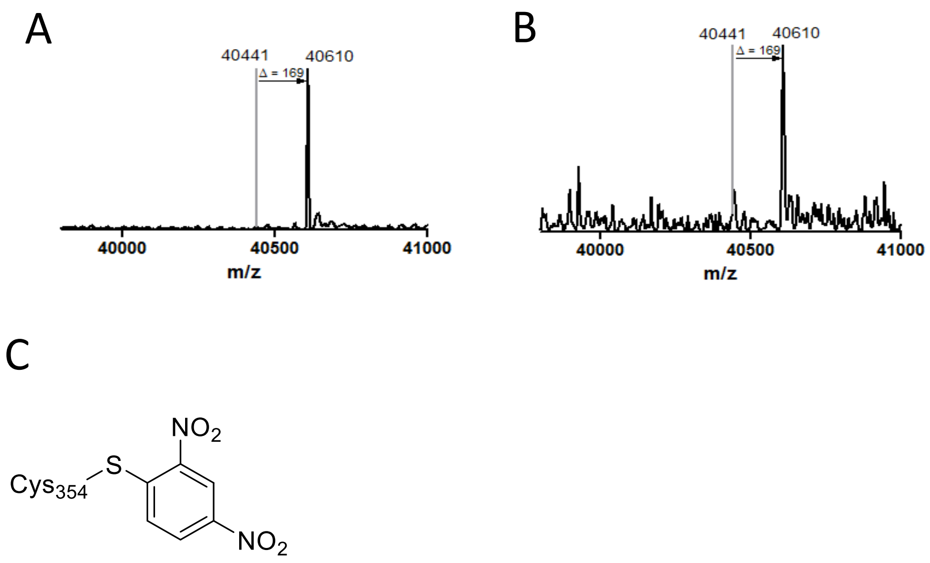

Figure S6. Mass spectra for Ldt_{Mt2} (1 μM) alone (grey) and in the presence of 100 μM probe (A) **2** and (B) **3** (black). (C) The mass difference of 169 Da corresponds to the addition of a dinitrophenyl group to the protein. The mass spectra were acquired after a 5 minute incubation. See Experimental Details for the conditions used.

A

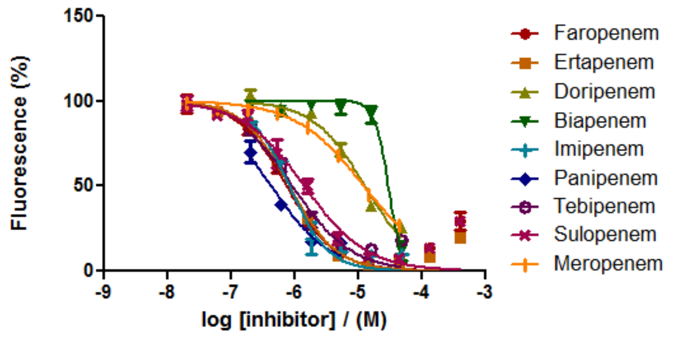
Fluorescence (%)
150
100
50
0
-9
-8
-7
-6
-5
-4
-3
log [inhibitor] / (M)
Faropenem
Ertapenem
Doripenem
Biapenem
Imipenem
Panipenem
Tebipenem
Sulopenem
Meropenem

B

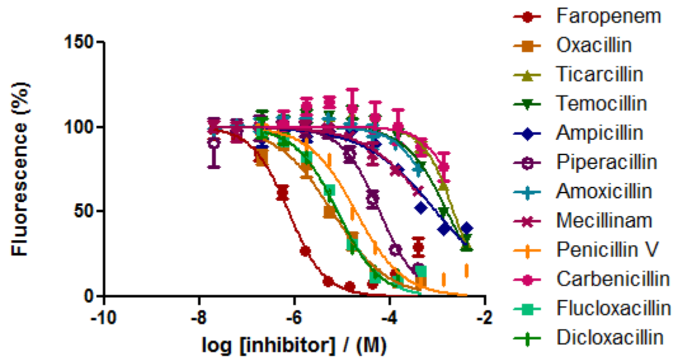
Fluorescence (%)
150
100
50
0
-10
-8
-6
-4
-2
log [inhibitor] / (M)
Faropenem
Oxacillin
Ticarcillin
Temocillin
Ampicillin
Piperacillin
Amoxicillin
Mecillinam
Penicillin V
Carbenicillin
Flucloxacillin
Dicloxacillin

C

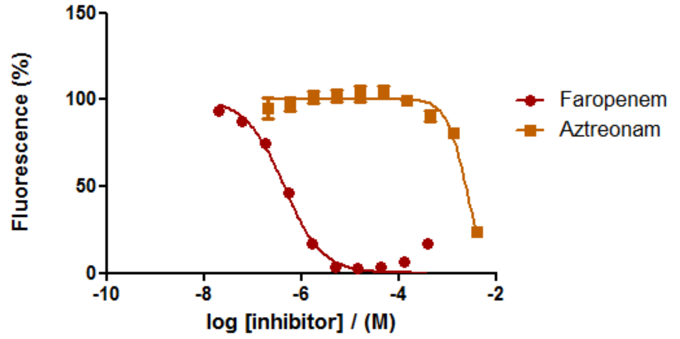
Fluorescence (%)
150
100
50
0
-10
-8
-6
-4
-2
log [inhibitor] / (M)
Faropenem
Aztreonam

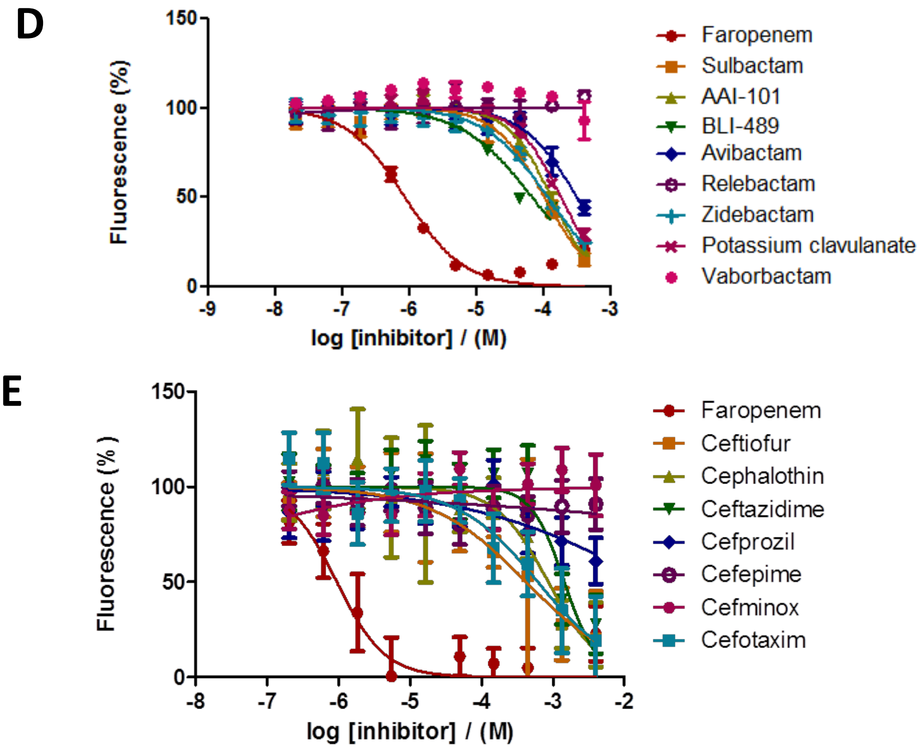

Figure S7. Dose response curves for (A) (carba)penems, (B) penicillins, (C), monobactam, (D) β-lactamase inhibitors and (E) cephalosporins with Ldt_{Mt2}. The curve for faropenem is shown in A-E for reference. Graphs A-D were obtained by applying the endpoint assay with fluorogenic probe **3**. Graph E was obtained by applying the kinetic assay with fluorogenic probe **2**. The data points shown represent the mean, with error bars representing the standard error in the mean (n = 4). See Experimental Details for the conditions used.

Table S1. IC_{50} and pIC_{50} values obtained from the dose-response analysis of the inhibitors with Ldt_{Mt2}, using the assay with fluorogenic probes **2** or **3** (errors represent SEM, n = 4).

**Compound**		**Dose-response analysis**	
**Name**	**Structure**	**IC_{50}**	**pIC_{50}**
Faropenem sodium hemipentahydrate	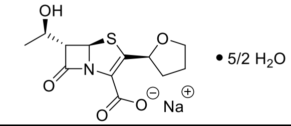	0.60 ± 0.01 μM	6.2 ± 0.1
Sulopenem	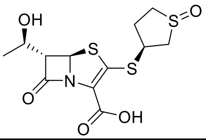	1.5 ± 0.1 μM	5.8 ± 0.1
Panipenem	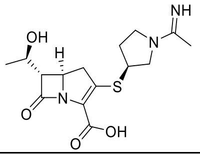	0.43 ± 0.05 μM	6.4 ± 0.1
Ertapenem sodium	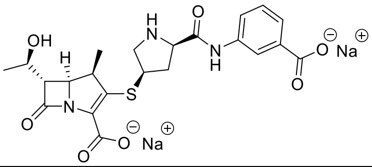	0.85 ± 0.09 μM	6.1 ± 0.1
Imipenem	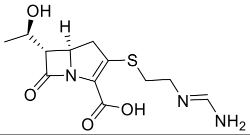	0.84 ± 0.08 μM	6.1 ± 0.1
Tebipenem	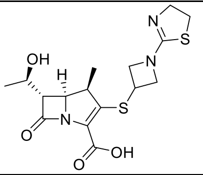	0.95 ± 0.04 μM	6.0 ± 0.1
Doripenem	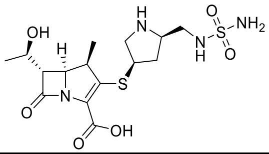	13 ± 1 μM	4.9 ± 0.1
Meropenem trihydrate	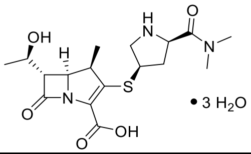	16 ± 1 μM	4.8 ± 0.1
Biapenem	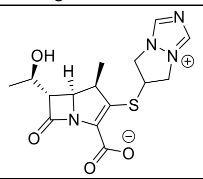	31 ± 2 μM	4.5 ± 0.1
Oxacillin sodium	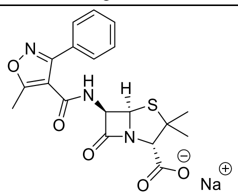	6.4 ± 0.8 μM	5.2 ± 0.1

Compound		Dose-response analysis	
**Name**	**Structure**	**IC_{50}**	**pIC_{50}**
Flucloxacillin sodium	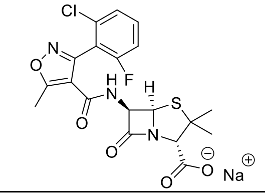	8.3 ± 0.8 μM	5.1 ± 0.1
Dicloxacillin sodium	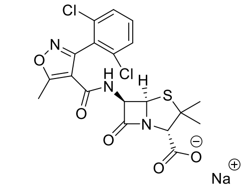	8.7 ± 0.6 μM	5.1 ± 0.1
Penicillin V potassium	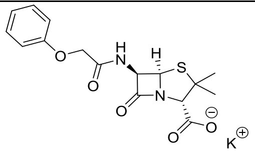	20 ± 2 μM	4.7 ± 0.1
Piperacillin sodium	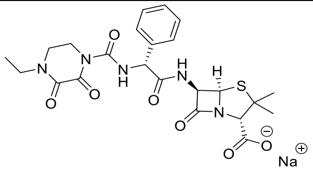	62 ± 7 μM	4.2 ± 0.1
Mecillinam	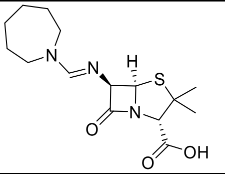	7.6 ± 0.1 mM	3.1 ± 0.1
Ampicillin	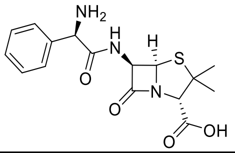	1.0 ± 0.2 mM	3.0 ± 0.1
Amoxicillin trihydrate	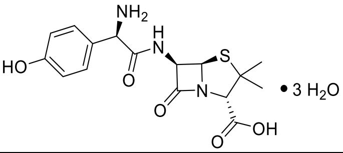	1.1 ± 0.4 mM	3.0 ± 0.1
Temocillin disodium	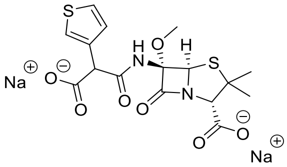	1.6 ± 0.2 mM	2.8 ± 0.1
Ticarcillin disodium	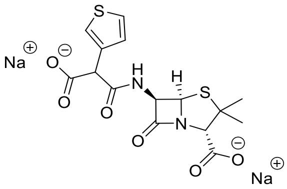	2.2 ± 0.2 mM	2.7 ± 0.1
Carbenicillin disodium	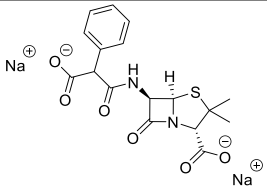	>4.0 mM	<2.4

Compound		Dose-response analysis	
**Name**	**Structure**	**IC_{50}**	**pIC_{50}**
Aztreonam	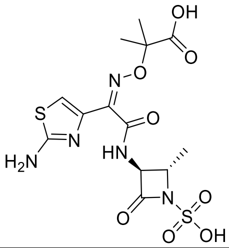	2.4 ± 0.2 mM	2.6 ± 0.1
Avibactam	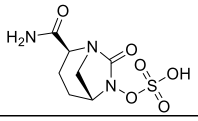	0.32 ± 0.04 mM	3.5 ± 0.1
Sulbactam	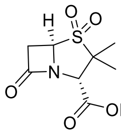	0.11 ± 0.01 mM	3.9 ± 0.1
AAI-101	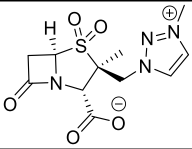	0.13 ± 0.01 mM	3.9 ± 0.1
Potassium clavulanate	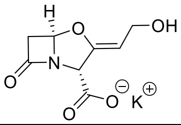	0.19 ± 0.01 mM	3.7 ± 0.1
BLI-489 hydrate	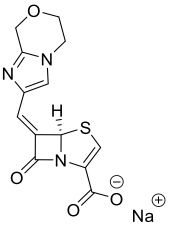	69 ± 10 µM	4.2 ± 0.1
Zidebactam	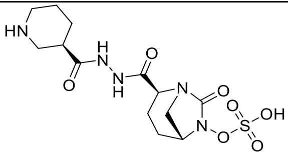	0.12 ± 0.01 mM	3.9 ± 0.1
Relebactam	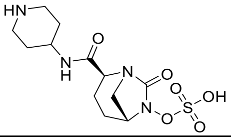	>4.0 mM	<2.4
Vaborbactam	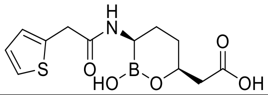	>4.0 mM	<2.4

Compound		Dose-response analysis	
**Name**	**Structure**	**IC_{50}**	**pIC_{50}**
Ceftiofur	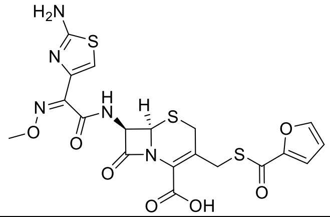	0.42 ± 0.07 mM	3.8 ± 0.1
Cefotaxime sodium salt	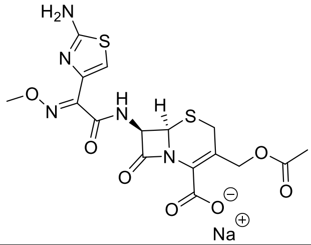	0.6 ± 0.3 mM	3.2 ± 0.1
Cephalothin sodium salt	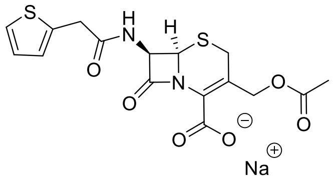	0.9 ± 0.3 mM	3.1 ± 0.1
Ceftazidime pentahydrate	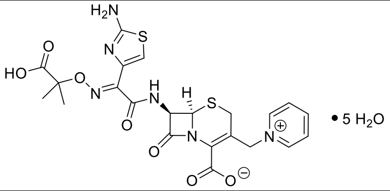	1.5 ± 0.5 mM	2.8 ± 0.1
Cefprozil	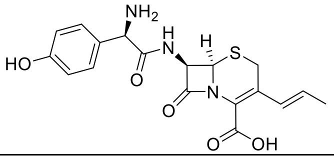	>4.0 mM	<2.4
Cefepime hydrochloride monohydrate	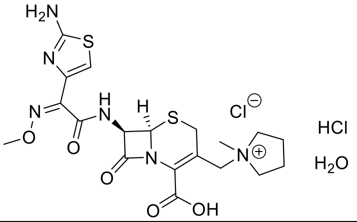	>4.0 mM	<2.4
Cefminox sodium salt	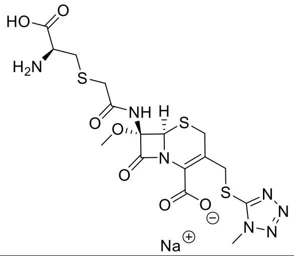	>4.0 mM	<2.4

References

1. Lohans, C. T.; Chan, H. T. H.; Malla, T. R.; Kumar, K.; Kamps, J.; McArdle, D. J. B.; van Groesen, E.; de Munnik, M.; Tooke, C. L.; Spencer, J.; Paton, R. S.; Brem, J.; Schofield, C. J., Non-Hydrolytic beta-Lactam Antibiotic Fragmentation by l,d-Transpeptidases and Serine beta-Lactamase Cysteine Variants. *Angew Chem Int Ed Engl* **2019,** *58* (7), 1990-1994.
2. Jiang, W.; Fu, Q.; Fan, H.; Ho, J.; Wang, W., A highly selective fluorescent probe for thiophenols. *Angew Chem Int Ed Engl* **2007,** *46* (44), 8445-8.
3. Maeda, H.; Matsuno, H.; Ushida, M.; Katayama, K.; Saeki, K.; Itoh, N., 2,4-Dinitrobenzenesulfonyl fluoresceins as fluorescent alternatives to Ellman's reagent in thiol-quantification enzyme assays. *Angew Chem Int Ed Engl* **2005,** *44* (19), 2922-5.
